# Dual-band polarimetric HRRP recognition via a brain-inspired multi-channel fusion feature extraction network

**DOI:** 10.3389/fnins.2023.1252179

**Published:** 2023-08-22

**Authors:** Wei Yang, Qiang Zhou, Mingchen Yuan, Yang Li, Yanhua Wang, Liang Zhang

**Affiliations:** ^1^Radar Research Laboratory, School of Information and Electronics, Beijing Institute of Technology, Beijing, China; ^2^Electromagnetic Sensing Research Center of CEMEE State Key Laboratory, School of Information and Electronics, Beijing Institute of Technology, Beijing, China; ^3^Chongqing Innovation Center, Beijing Institute of Technology, Chongqing, China; ^4^Beijing Key Laboratory of Embedded Real-time Information Processing Technology, Beijing, China; ^5^Advanced Technology Research Institute, Beijing Institute of Technology, Jinan, Shandong, China

**Keywords:** radar automatic target recognition (RATR), dual-band polarimetric HRRP, information fusion, brain-inspired neural network, attention mechanism

## Abstract

Radar high-resolution range profile (HRRP) provides geometric and structural information of target, which is important for radar automatic target recognition (RATR). However, due to the limited information dimension of HRRP, achieving accurate target recognition is challenging in applications. In recent years, with the rapid development of radar components and signal processing technology, the acquisition and use of target multi-frequency and polarization scattering information has become a significant way to improve target recognition performance. Meanwhile, deep learning inspired by the human brain has shown great promise in pattern recognition applications. In this paper, a Multi-channel Fusion Feature Extraction Network (MFFE-Net) inspired by the human brain is proposed for dual-band polarimetric HRRP, aiming at addressing the challenges faced in HRRP target recognition. In the proposed network, inspired by the human brain’s multi-dimensional information interaction, the similarity and difference features of dual-frequency HRRP are first extracted to realize the interactive fusion of frequency features. Then, inspired by the human brain’s selective attention mechanism, the interactive weights are obtained for multi-polarization features and multi-scale representation, enabling feature aggregation and multi-scale fusion. Finally, inspired by the human brain’s hierarchical learning mechanism, the layer-by-layer feature extraction and fusion with residual connections are designed to enhance the separability of features. Experiments on simulated and measured datasets verify the accurate recognition capability of MFFE-Net, and ablative studies are conducted to confirm the effectiveness of components of network for recognition.

## 1. Introduction

Brain-inspired computing is inspired by the human brain, which utilizes multiple types of information, such as visual, sound, and tactus, simultaneously to deal with tasks. Through interactions among various neural systems or neurons, the brain is capable of integrating diverse information while focusing on key elements (Muttenthaler et al., 2020). This information processing approach of the brain has inspired the development of neural network-based multidimensional data fusion techniques (LeCun et al., 2015), such as target detection (Yuan et al., 2023), tracking (Han et al., 2019a,^2022^), and recognition (Zeng et al., 2022b). By studying the information processing mechanisms of the human brain, networks can enhance their understanding of objects and improve confidence in decision-making.

Radar target High-resolution range profile (HRRP) represents the distribution of scattering centers along radar line of sight (LOS), providing the geometric and structural characteristics of a target (Chen et al., 2022). Because of its convenient acquisition, processing, and storage (Wang et al., 2021), it plays an important role in RATR. However, due to the limited information dimension of HRRP, it is difficult to accurately distinguish targets in complex electromagnetic environments. In recent years, with the rapid advancement of radar components and signal processing technology, acquiring target multi-frequency bands and polarization scattering information has become an important development direction to improve the target recognition performance of HRRP in complex environments (Shi et al., 2020).

In recent decades, several works aiming at fusing and utilizing multidimensional radar data, which include multi-frequency and multi-polarization echoes to improve recognition performance, have been investigated. To exploit the complementary information, data-level fusion is an important technical approach (Zhang, 2010). It involves correlation registration of raw data and fusion based on signal processing algorithms, including fuzzy parameter estimation theory (Solaiman et al., 1999), Markovian model (Fouque et al., 2000), wavelet transform (Cakir et al., 1999), jointly modeling (Han et al., 2017, 2019b), and other algorithms. Ruohong et al. (2010) fused multiple SAR images using principal component analysis and discrete wavelet transform, followed by SVM classification. Furthermore, a fusion approach combining C, L-band, and multi-polarization (HH, HV, and VV) Synthetic Aperture Radar (SAR) images through discrete wavelet transform was utilized, and recognition was performed based on polarization feature vectors (Hong et al., 2002). Chang et al. (2004) utilized the feature scale uniformity transform to combine the relevant features from hyperspectral and SAR data sources, followed by an optimal Boolean classifier, which significantly enhances classification accuracy compared to a single source.

Feature-level fusion is also important for multidimensional data fusion, which involves extracting representative features from different data sources and integrating them into a comprehensive feature representation (Meng et al., 2020). Features mainly include manual and deep features, while fusion includes concatenation, addition, dimension transformation, and more. In Jiang et al. (2022), multiple high-resolution features were extracted from HRRPs, which fused with a CNN to improve unmanned aerial vehicle (UAV) recognition. Considering the temporal dependencies and multi-domain features within HRRPs, Zeng et al. (2022a) proposed the Multi-Input Convolutional Gated Recurrent Unit (MIConvGRU) structure, which utilizes temporal, frequency, and time-domain information for recognition. Furthermore, there exist studies that leverage a combination of physical knowledge, attention mechanism, and deep networks (Zhang L. et al., 2020; Pan et al., 2021; Liu et al., 2022). Zhang and Zhang (2022), used self-attention to weight and interactively concatenate different polarization channels. Zhang et al. (2021) incorporated artificial features by attention to guide the model’s focus on HRRP units with richer scattering information.

Decision-level fusion involves analyzing and integrating multiple decision results to improve system robustness (Sinha et al., 2008), mainly including voting, Dempster-Shafer (D-S) evidence theory (Shao et al., 2016; Qin et al., 2022), Bayesian estimation (Huan et al., 2010; Du et al., 2012; Wei et al., 2015), and expert rule. Shengqi et al. (2015), proposed a Joint Sparse Representation (JSR) method for multi-polarization HRRP recognition. Each single-polarization HRRP is represented by adaptively selected atoms from its corresponding dictionary, while recognition is conducted by using an overall minimum reconstruction residual criterion. In the study by Liu and Li (2013), the decisions of SAR were made using a Fast Sparse Representation Classifier and a Support Vector Machine Classifier; the decision results were then fused according to Bayesian rules. For HRRP recognition, Zhang et al. (2011) fused the decision results of multiple classifiers using weighted voting.

Although the approaches above enhance fusion performance, there are still two issues that need to be addressed in multidimensional data fusion and recognition:

(1) Feature extraction within a single dimension: Current research mainly focuses on feature extraction within individual polarization or frequency channels, lacking exploration of the correlations between different channels. This lack of investigation fails to ensure the robustness of feature extraction within frequency or polarization dimensions and the effectiveness of subsequent fusion.
(2) Feature fusion between multidimensional data: Due to the different emphases of target discrimination information contained in multidimensional data, feature fusion is necessary for better utilizing complementary information. However, there is little research on the fusion of multi-frequency polarization HRRPs. Existing methods mainly involve concatenating or summing multidimensional features, without fully fusing the information reflecting the variations of target characteristics across frequency or polarization. If this crucial information is focused on during the fusion stage, the recognition performance can be greatly improved.

In order to address the aforementioned issues, this paper proposes a brain-inspired multi-channel interaction feature extraction network for dual-frequency polarization HRRP fusion recognition, aiming to improve HRRP target recognition performance in complex environments. The proposed network is inspired by the information processing mechanism of the human brain, so as to have more effective multi-dimensional information fusion and feature representation capabilities. First, we design a Dual-Frequency Information Fusion (DFIF) module which utilizes the Siamese network and attention mechanism to extract similar and different scattering center features between frequencies. Second, we design a Multi-Polarization Information Fusion (MPIF) module that aggregates the multi-polarization features through a dual-attention mechanism, and conduct multi-scale polarization feature fusion with a symmetric encoder-decoder structure. Third, we design a Residual Feature Enhancement Learning (RFEL) module that enhances the separability of features through residual-based learning and a progressive learning structure. Moreover, a novel hybrid loss function, consisting of scatter center loss, maximum coding rate reduction loss, and cross-entropy loss, is introduced to reinforce feature extraction and fusion. In general, the main contributions of this paper are as follows:

(1) Inspired by the interaction of multi-dimensional information mechanism in the human brain, we propose a Dual-Frequency Information Fusion (DFIF) module that utilizes the Siamese network and attention mechanism to extract similar scattering center features and extract the differential scattering center features through subtraction and adaptively weighting between dual frequencies. With this module, the dual-frequency features can be extracted and fused effectively.
(2) Inspired by the attention mechanism in the human brain, we propose a Multi-polarization Information Fusion (MPIF) module that uses the double attention mechanism to identify the key feature as generates global descriptor, then assign them to each feature location to realize the multi-polarization feature aggregation. And we fuse the multi-scale fusion with a symmetric encoder-decoder structure.
(3) Inspired by the human brain’s hierarchical learning mechanism, we design a Residual feature enhancement learning (RFEL) module to perform layer-by-layer feature extraction and fusion with residual connections, which can enhance the separability of features.

The rest of the paper is as follows: Section “2. Proposed method” describes the proposed MFFE-Net in detail. Section “3. Experimental results and analysis” analyzes and evaluates the performance of MFFE-Net. Section “4. Conclusion” concludes this paper.

## 2. Proposed method

This part first provides an overview of the proposed MFFE-Net. Then, the sub modules of MFFE-Net are introduced, respectively. Finally, we introduce the loss function we designed.

### 2.1. Overview of proposed MFFE-Net

**FIGURE 1:**
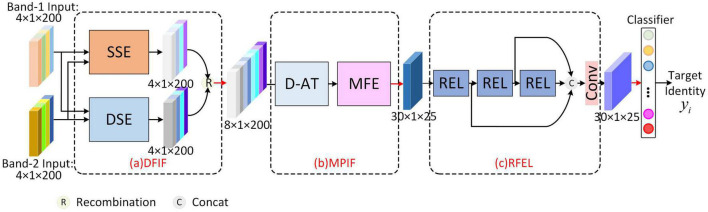
Overview of the proposed MFFE-Net. **(a)** Dual-frequency Information Fusion module. **(b)** Multi-polarization Information Fusion module. **(c)** Residual Feature Enhancement Learning module.

The overall structure of MFFE-Net is shown in Figure 1, which is a cascading structure. First, the Dual-Frequency Information Fusion (DFIF) module extracts similar scattering center features and the differential scattering center feature of dual-frequency HRRP, respectively by Similarity Scattering center feature Extraction (SSE) block and Differential Scattering center feature Extraction (DSE) block. Second, the frequency fusion features are sent into multi-polarization information fusion (MPIF) module and consecutively pass-through the Double Attention aggregation (D-AT) block and Multi-scale Feature Extraction (MFE) block to achieve the aggregation representation of multi-polarization information and the fusion of multi-scale features. Third, the separability of fusion features is enhanced through the Residual Enhancement Learning (REL) unit of Residual Feature Enhancement Learning (RFEL) module. Finally, features are fed into the classifier to obtain the final classification.

Moreover, the network is trained and updated by a hybrid loss consisting of scattering center loss, maximum coding rate decline loss, and cross-entropy loss, which, respectively act on DFIF, RFEL, and Classifier.

### 2.2. Dual-frequency Information Fusion module

The human brain is capable of interacting with visual, taste, tactile, and other sensory information based on certain criteria, enhancing the expression of features and thereby improving its understanding of things (Ji et al., 2023), which can guide deep learning-based radar multi-dimensional information processing. Drawing inspiration from the human brain’s multi-dimensional information interaction, we proposed a Dual-frequency Information Fusion module, depicted in Figure 1a, which aims to mine frequency-dimensional features from two aspects of scattering center similarity and difference through the means of feature extraction. For the first aspect, we propose an SSE Block based on the idea of the Siamese network, which employs convolution layers with shared parameters to extract similarity scattering center features, and then add them by attention weighting. For the second aspect, we designed a DSE Block for differential scattering center feature extraction. Specifically, we searched for the differential scattering center through subtraction, and then enhanced the differential scattering center features through spatial attention and a convolution layer. Below, we will discuss the specifics of these two submodules.

#### 2.2.1. Similarity scattering center feature extraction block

**FIGURE 2:**
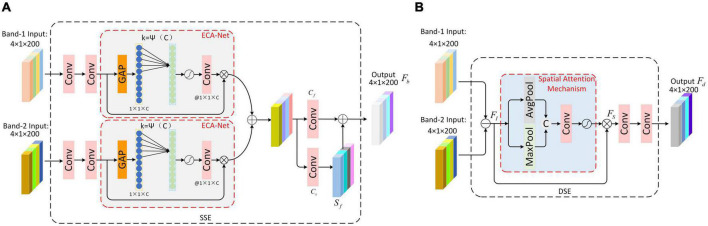
Dual-frequency Information Fusion module. **(A)** The structure of Similarity Scattering center feature Extraction block (SSE) **(B)** Differential Scattering Center Feature Extraction Block.

The SSE Block is shown in Figure 2A. First, the dual-frequency HRRPs are fed into the SSE. Each frequency HRRP is processed by convolutional layers to extract the scattering center features. Inspired by the Siamese network, the convolutional layer parameters are shared across two routes to obtain similar scattering center features. Second, the dual-frequency similarity scattering center features are weighted adaptively using the channel attention module. With an aim to preserve the integrity of scattering center features in different frequency HRRPs, compared to the traditional SE-Net, ECA-Net avoids dimensionality reduction and effectively captures inter-channel interaction information, so we introduce ECA-Net (Wang et al., 2020) to achieve adaptive weighting. Set *F* = [*f*_1_, *f*_2_, …*f*_*C*_], *f*_*i*_ ∈ *R*^*L*×1^, *i* = 1, 2, …*C* to denote the input feature maps to ECA-Net, their global spatial information is squeezed through the global average pooling, which is used as the channel descriptor of the feature map. Then, 1*k convolution is used to realize cross-channel information interaction. Given the channel dimension C, the convolution kernel size *k* can be adaptively determined as:


(1) $$k=\psi \,\left(C\right)={\left|\log_{2}\left(C\right)\,/\,\gamma +b\,/\,\gamma \right|}_{o\,d\,d}$$

where |*t*|_*odd*_ represents the odd number closest to *t*. We refer to the experimental setup in Wang et al. (2020):γ = 2, *b* = 1.

After that, the scattering center features with adaptive weighting of dual-frequency bands are added and fused to obtain the preliminary fusion result *F_*b*0_*.

Finally, we further extract the feature *F_*b*0_* of the previous step; specifically, we pass it through two parallel convolution layers, wherein the output of convolution layer *C*_*f*_ is similarity scattering center feature *F*_*b*_, while the convolution layer *C*_*s*_ extracts the robust scattering center feature *S*_*f*_, which is constrained by the scattering center loss function from the perspective of backpropagation, reducing the influence of other regions on the scattering centers. Finally, the robust scattering center feature *S*_*f*_ is superimposed onto *F*_*b*_. This process yields the enhanced similarity scattering center features, which is outlined as follows:


(2) $$S_{f}=C_{s}\,\left(F_{b\,0}\right)$$

(3) $$F_{b}=C_{f}\,\left(F_{b\,0}\right)+S_{f}$$

#### 2.2.2. Differential scattering center feature extraction block

The DSE Block is shown in Figure 2B. Firstly, the dual-frequency HRRPs are subtracted to obtain the differential scattering center information *F*_*I*_. Second, a spatial attention module is designed to dynamically search for scattering center differential information that contribute to recognition, which applies average pooling and maximum pooling operations along the channel axis and connects them to generate an effective feature description. Then, the feature description is sent into a convolutional layer to generate a final spatial attention weight sequence through an activation function, which is then multiplied with *F*_*I*_ to obtain the attention-weighted feature *F*_*S*_. Third, the differential scattering center features *F*_*d*_ are obtained by further feature extraction using the convolution layer.

Additionally, the similar scattering center features from SSE and the different scattering center features from DSE are recombined. By reassembling the corresponding channel neighbors, the dual-frequency fusion features are obtained.

### 2.3. Multi-polarization Information Fusion module

In visual tasks, the human brain can selectively focus on specific information while ignoring other irrelevant information, and dynamically adjusts the focus of attention (Shi et al., 2022). This helps reduce the cognitive load, allowing the brain to process complex environments and stimuli. Inspired by the human brain’s selective attention mechanism, for the polarization dimension, our approach involves first aggregating the polarization information and subsequently conducting multi-scale feature extraction and fusion. This paper proposes a multi-polarization information fusion (MPIF) module, which consists of a dual attention aggregation (D-AT) block and multi-scale feature extraction (MFE) block. First, the previous module’s output feature is initially processed by D-AT, which identifies the key feature and generates global descriptors; it then assigns them to each feature location to realize the multi-polarization feature aggregation. Second, the aggregate features are fed into the MFE block to realize multi-scale feature extraction and fusion through the symmetric encode-decode structure. The D-AT and MFE are described separately below.

#### 2.3.1. Double-attention aggregation block

**FIGURE 3:**
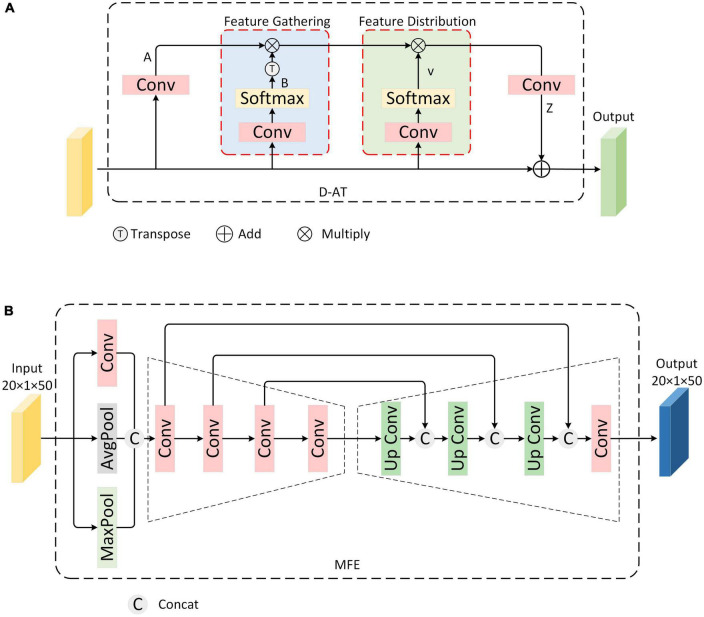
Multi-polarization Information Fusion module **(A)** Structure of Double-Attention Aggregation block **(B)** Multi-scale feature extraction block.

Our goal is to aggregate all the features through an attention mechanism, thereby obtaining the weights of key features, and subsequently reassigning these weights to them. Therefore, we choose the dual attention aggregation (D-AT) block (Chen et al., 2018), which is shown in Figure 3A. First, it extracts features A and B by convoluting the input feature maps. Then the outer product of the vectors in the two feature graphs A and B is taken, that is, the matrix multiplication of A and B:


(4) A=C⁢o⁢n⁢v⁢1⁢(X)

(5) B=s⁢o⁢f⁢t⁢m⁢a⁢x⁢(C⁢o⁢n⁢v⁢2⁢(X))

(6) $$G\,\left(A,B\right)=A\,B^{T}=\left[g_{1},\ldots ,g_{n}\right]\in {\mathbb{R}}^{m\times n},g_{i}=A\,{\bar{b}}_{i}^{T}=\sum_{\forall }{\bar{b}}_{i\,j}\,a_{j}$$

where, *B* is ensured by SoftMax after convolution to $\sum_{j}{\bar{b}}_{i\,j}=1$, making it a valid attention weight vector. *G* can be understood as the output of a set of key feature primitives, each *g*_*i*_ is obtained by aggregating local features weighted by $\bar{b_{i}}$.

Second, features obtained from the first step are distributed across each location of input space, distributing an adaptive primitive for the need of each location’s feature *v*_*i*_ to capture more complex relationships. The implementation can be seen as selecting a subset of feature vectors from *G*_*gather*_*(X)* using a soft focus:


(7) $$z_{i}=\sum_{\forall }v_{i\,j}\,g_{j}=G_{g\,a\,t\,h\,e\,r}\,\left(X\right)\,v_{i}=G\,v_{i},w\,h\,e\,r\,e\,\sum_{\forall i\,j}v_{i\,j}=1$$

Finally, an additional convolution layer is added at the end to extend the number of channels for the output *Z*, enabling it to be encoded back into the input *X* by adding elements. The general formula is as follows:


(8) $$\begin{array}{c} Z^{\prime }=C\,o\,n\,v\,4\,\left(F_{d\,i\,s\,t\,r}\,\left(G_{g\,a\,t\,h\,e\,r}\,\left(X\right),V\right)\right)\\ =C\,o\,n\,v\,4\,\left(G_{g\,a\,t\,h\,e\,r}\,\left(X\right)\,s\,o\,f\,t\,m\,a\,x\,\left(C\,o\,n\,v\,3\,\left(X\right)\right)\right)\\ =C\,o\,n\,v\,4\,\left(\left[C\,o\,n\,v\,1\,\left(X\right)\,s\,o\,f\,t\,m\,a\,x\,\left(C\,o\,n\,v\,2\,\left(X\right)\right)\right]\,s\,o\,f\,t\,m\,a\,x\,\left(C\,o\,n\,v\,3\,\left(X\right)\right)\right) \end{array}$$

The D-AT block realizes the aggregation of multi-polarization features, which serves as the next step of polarization feature fusion.

#### 2.3.2. Multi-scale feature extraction block

The multi-scale feature fusion block is shown in Figure 3B. Specifically, a convolutional autoencoder structure is designed to fuse them. Convolution has local awareness of feature maps, while average pooling and maximum pooling compress feature maps based on mean and maximum, respectively. First, to enrich the representation of polarization dimension information, the features from D-AT are extracted through the convolution layer, average pooling layer, and maximum pooling layer, respectively. Second, considering extracting polarization features from different scales and that low-level features tend to capture details and high-level features encapsulate overall characteristics, a convolutional encoder structure to obtain features of multiple scales through convolution operations of different sizes is used. Finally, MFE block fuses the multi-scale features obtained in the previous step. Specifically, the convolutional decoder structure is designed to retrieve and reconstruct the output features of the last layer of the encoder. Simultaneously, skip connections are utilized to splice and fuse the features from each layer of the decoder with the corresponding scale features from the encoder. After that, the final fusion result is obtained through the convolution layer.

### 2.4. Residual feature enhancement learning module

**FIGURE 4:**
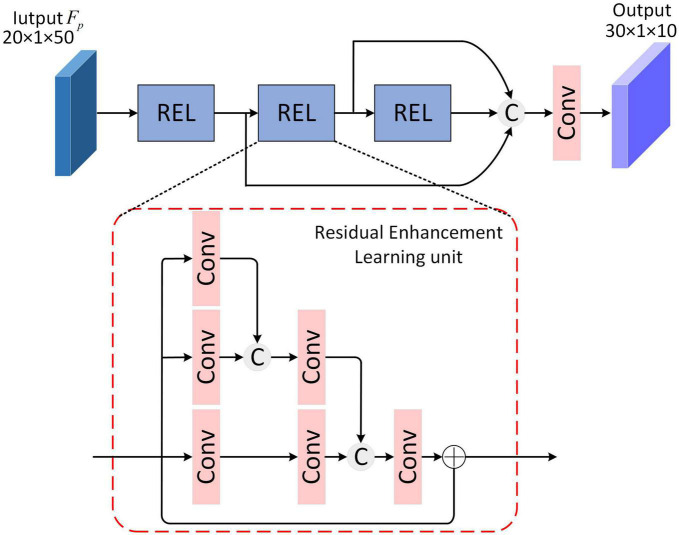
Structure of Residual Feature Enhancement Learning module.

The brain’s hierarchical learning mechanism refers to the process of gradually establishing complex hierarchical structures, from low-level perception to high-level abstraction, to progressively learn and comprehend information. This mechanism enables the brain to process information at different levels, leading to comprehensive and profound cognition (Ji et al., 2022). Inspired by the human brain’s hierarchical learning mechanism, to further enhance feature separability and obtain the most effective linear discriminant representation for target recognition, the Residual Feature Enhancement Learning (RFEL) module is designed, depicted in Figure 4. For a Residual Enhancement learning (REL) unit in RFEL, features are extracted through three parallel convolutional routes, in which the convolutional layer is increased step-by-step, and the features from the upper level are joined with the features of the current level, and then passed on to the subsequent convolutional layer within the current level. This structure is considered feasible in radar target recognition and has been experimentally validated for its effectiveness (Pei et al., 2017). Moreover, the feature separability is enhanced by adding the initial feature to the final convolution result by skip-connection. This integration further improves the discriminant representation of the features. In addition to REL, skip-connections are also adopted, which splices the features learned by multiple REL, strengthens the transmission of features, and reduces the number of model parameters to a certain extent.

### 2.5. Loss function

To sum up, the mixed loss function used in the model of the paper is:


(9) $$L_{a\,l\,l}=\alpha \,L_{s}+\beta \,L_{\Delta \,R}+\gamma \,L_{c}$$

The *L*_*s*_ represents scattering center loss, *L*_△ *R*_ represents maximum coding rate reduction loss, and *L*_*c*_ represents crossentropy loss. α,β, and γ are the weights coefficient of scattering loss, MCR2 loss, and cross entropy loss, respectively.

To improve the effectiveness of dual-frequency similarity scattering center feature fusion, a scattering center loss function is proposed, which is defined as follows:


(10) $$L_{s}=L_{s\,n}+L_{s\,l}$$

(11) $$L_{s\,n}={||S_{f\,n}-\left(X_{s\,n}^{1}\cup X_{s\,n}^{2}\,\ldots \cup X_{s\,n}^{x}\right)||}_{2}$$

(12) $$L_{s\,l}={||S_{f\,l}-\left(X_{s\,l}^{1}\cup X_{s\,l}^{2}\,\ldots \cup X_{s\,l}^{x}\right)||}_{2}$$

Where *L*_*s*_ is the loss of scattering center, *L*_*sn*_ is the loss of the number of scattering centers, and *L*_*sl*_ is the loss of the location of scattering centers. *S*_*fn*_ is the information about the number of fused HRRP scattering centers, and *X*^i^*_*sn*_* is the information about the number of HRRP scattering centers in the *i-th* frequency. *S*_*fn*_ is the location information of HRRP scattering center, and *X*^i^*_*sl*_* is the location information of HRRP scattering center in the *i-th* frequency. For *S*_*fn*_ and *S*_*fl*_, they are obtained from the sequence of HRRP strong scattering centers *S*_*f*_ extracted by the convolution layer *C*_*s*_ of SSE.

Furthermore, in the RFEL module, we employed a maximum coding rate reduction (MCR2) loss function to constrain the feature enhancement effect from the perspective of backpropagation. This loss function achieves the compression of intra-class distances in the feature space and expands the overall space, thereby enhancing feature separability. The RFEL module and the MCR2 loss function complement each other (Ma et al., 2007; Wu et al., 2021; Chan et al., 2022). The MCR2 loss function is depicted as follows:


(13) $$L_{\Delta \,R}=\Delta \,R\,\left(Z,\Pi ,\varepsilon \right)=R\,\left(Z,\varepsilon \right)-R_{c}\,\left(Z,\varepsilon |\Pi \right)$$

$$=\underset{R\,\left(Z,\varepsilon \right)}{\underbrace{\frac{1}{2}\,\log\text{det}\,\left(I+\alpha \,Z\,Z^{*}\right)}}-\underset{R_{c}\,\left(Z,\varepsilon |\Pi \right)}{\underbrace{\sum_{j=1}^{k}\frac{\gamma_{j}}{2}\,\log\text{det}\,\left(I+\alpha_{j}\,Z\,\Pi^{j}\,Z^{*}\right)}}$$

Where Δ*R*(*Z*,Π,ε) represents the change of encoding rate, and *I* is the identity matrix. Z = [*z*^1^,…,*z^m^*], *z^i^*ε*R,i* = 1, …,*m* is the given feature set, *Z* contains *k* categories,*Z* = *Z*^1^ ⋃ *Z*^2^ ⋃ … ⋃ *Z^k^*, α = *n*/*m*^2^,α_*j*_ = *n*/*tr* (∏^*j*^)^2^, and γ_*j*_ = *tr* (∏^*j*^) /*m*, for *j* = *1*,…,*k*, $\prod ={\left\{\prod^{j}\in {\mathbb{R}}^{m\times m}\right\}}_{j=1}^{k}$ is a set of diagonal matrices, the diagonal term ∏*^j^*(*i,i*) of ∏*^j^* represents the probability that sample *z^i^* belongs to subset *j*.

Finally, the cross-entropy loss function is used for classification.

## 3. Experimental results and analysis

This section validates the effectiveness of our proposed model using dual-frequency and multi-polarization HRRPs data. Section “3.1. Data description” introduces the simulation dataset and the measured dataset. Section “3.2. Experiment settings” presents the comparison methods employed in the experiments, along with the configuration of MFFE-Net and the experimental conditions. Sections “3.3. Recognition results” presents the experimental results obtained from the simulation dataset and the measured dataset, respectively. In section “3.4. Ablation study and analysis,” ablation experiments were conducted to show the feature visualization of our model and analyze the results regarding the MCR2 loss effect. This analysis serves to further validate the effectiveness of our proposed method.

### 3.1. Data description

#### 3.1.1. Electromagnetic simulation dataset of five Civilian Vehicles (ESD)

**FIGURE 5:**
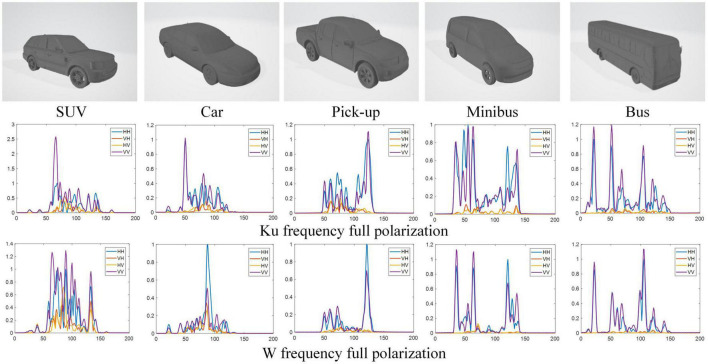
Simulation target models and full polarimetric HRRP samples of five different vehicles.

We constructed a dual-frequency multi-polarization simulation dataset, which considered a complex identification scenario. The HRRP data of Ku(16 GHz)/W(92 GHz)-center frequency with 0.75 GHz bandwidth, and full polarization (HH, VH, HV, and VV), included five classes of civilian vehicle targets, namely, car, SUV, pick-up, minibus, and bus. The simulation uses the target CAD model with 60 azimuth angles of [1°, 360°] spaced by 6°, with elevation angle of 28°, 30°. The HRRP of each azimuth is enhanced with noise based on the Monte Carlo method, and 30 HRRP samples are obtained. As a result, the dataset contains five targets for a total of 18,000 (*18,000* = *5* × *60* × *30* × *2*, *target number* × *azimuth angle number* × *data augmentation number* × *elevation angle number*) dual-frequency and multi-polarization HRRP samples. The simulation target models and the HRRPs are shown in Figure 5 (only the fully polarization HRRPs of 0° azimuth is shown). Based on the collected data, we conduct our experiments under two conditions. In the first condition, we randomly select 70% of the samples with elevation angle of 30° for training and 30% for testing, which results in 6,300 samples of training set and 2,700 samples of test set. In the second condition, in order to test the robustness of the proposed model, we selected data with an elevation angle of 30° as training set and data with an elevation angle of 28° as test set, which results in 9,000 samples of training set and 9,000 samples of test set.

#### 3.1.2. Real measurement dataset of three Civilian Vehicles (RMD)

**FIGURE 6:**
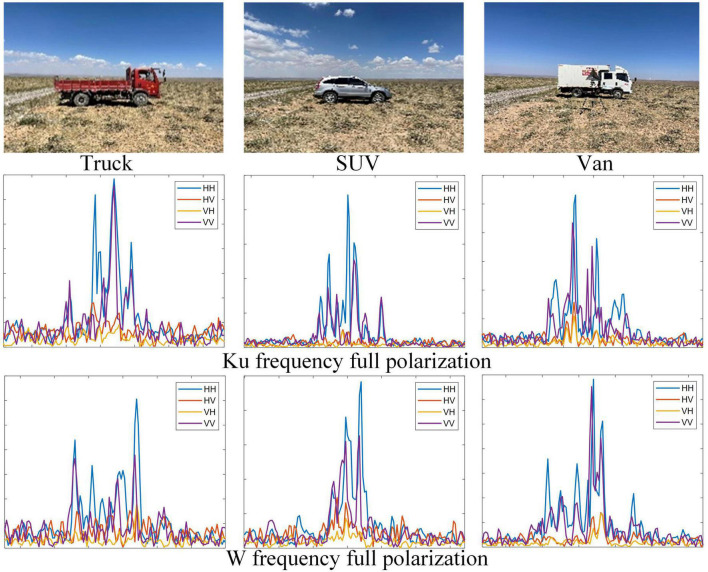
Real target models and full polarimetric HRRP samples of three different vehicles.

The measured data set includes three common types of vehicles: truck, SUV, and van. The radar operates at Ku(16 GHz) and W(92 GHz) center frequency, and bandwidth is 1.25 GHz. In the outfield scene, the measured data is collected discontinuously for stationary targets. For each type of vehicle target, dual-frequency and full-polarization HRRPs are collected with 8 azimuth angles of [1°,360°] spaced by 45°. HRRPs of the three targets are shown in Figure 6 (only HRRPs of Head attitude are shown). After processing, we obtained a total of 7,200 frames of dual-frequency full-polarization HRRPs, of which trucks, SUVs, and crates each have 2,400 frames. We randomly sampled 70% of HRRP samples from all the data for training, and the remaining 30% samples were used for testing.

### 3.2. Experiment settings

#### 3.2.1. Comparison method

We compare the performance of MFFE-Net with traditional target recognition methods by adjusting these methods to suit the dual-frequency full polarization data. Specifically, we consider machine learning methods including Support Vector Machine (SVM) (Lardeux et al., 2006) and K-Nearest Neighbor (KNN) (Wenbo et al., 2019), neural networks such as One-dimensional Convolutional Neural Network (1D-CNN) (Song et al., 2019), LSTM Recurrent Neural Network (LSTMRNN) (Jithesh et al., 2017), and stacked autoencoders (SAE) (Zhao et al., 2018), one-dimensional stack convolutional autoencoders (1D-SCAE) (Zhang G. et al., 2020), VGGNet (Jun et al., 2018), ResNet-34 (Park et al., 2020), and DenseNet (Du et al., 2021).

Specifically, for SVM, KNN, AE, and LSTM, we splice the HRRP sequence of eight dual-frequency fully polarized channels in the distance dimension to form an input 1D-tensor of *1* × (1*8*N) (*N* is the number of HRRP distance units). For CNN, CAE, VGGNet, ResNet-34, and DenseNet, we splice multi-frequency and multi-polarization HRRP in channel dimension to obtain *8* × *N* input tensor. The remaining experimental conditions are consistent with MFFE-Net.

#### 3.2.2. Network configuration

The specific configuration of the proposed model (Take the model using dual band full polarization as an example) is shown in Table 1, where Conv represents Convolutional layer, their hyper-parameters denote as (number of input feature) @ (kernel size of Conv). *Lx_#y* represents the *y-th* branch of *x-th* layer. *M* represents the number of targets. The size of output is expressed as *Channel* × *H* × *W* (for simulation HRRP, *H* = 1, *W* = 200).

**TABLE 1:** Detailed configurations of MFFE-Net.

Module		Layer		Configuration	Output size
Dual-frequency information fusion module	SSE	L1_#1,#2	Conv	4@1 × 3	4 × 1 × 200
		L2_#1,#2	Conv	4@1 × 3	4 × 1 × 200
		L3_#1,#2	ECA-Net	4@1 × 7	4 × 1 × 200
		L4_#1,#2	Conv	4@1 × 7	4 × 1 × 200
	DSE	L1	SAM		4 × 1 × 200
		L2	Conv	4@1 × 3	4 × 1 × 200
		L3	Conv	4@1 × 3	4 × 1 × 200
Multi-polarization information fusion module	D-AT	L1,2,3,4	Conv	8@1 × 1	8 × 1 × 200
	MPF	L1_#1	Conv	8@1 × 3	8 × 1 × 200
		L1_#2	AvgPool		8 × 1 × 200
		L1_#3	MaxPool		8 × 1 × 200
		L4,5,6,7	Conv	8@1 × 5	10 × 1 × 25
		L8,9,10	Up Conv	8@1 × 4	40 × 1 × 200
		L11	Conv	8@1 × 8	30 × 1 × 25
Residual feature enhancement learning module	REL1,2,3	L1_#1,#2	Conv	30@1 × 5	15 × 1 × 25
		L1_#3	Conv	30@1 × 5	30 × 1 × 25
		L2_#1,#2	Conv	30@1 × 5	15 × 1 × 25
		L3	Conv	30@1 × 5	30 × 1 × 25
Classification	FC	L1	FC layer	1,000	500
		L2	FC layer	100	M
		L3	Softmax	M	

#### 3.2.3. Experimental conditions

To quantitatively evaluate the performance of each model, we utilize several metrics including overall accuracy (OA) and per-class accuracy (PA), F1-Score, and AUC. Furthermore, all experiments are conducted using PyTorch codes on a 64-bit Linux operating system equipped with 24 GB RAM and 2 NVIDIA GeForce RTX 3,090 graphics cards. In the training phase, the batch size is set to 32, the learning rate is 0.01 with the decay of 0.95 times per epoch, and the network is optimized with adaptive moment estimation (Adam) algorithm.

### 3.3. Recognition results

#### 3.3.1. Experimental results on ESD

This paper conducted a comparison between MFFE-Net and traditional target recognition methods to assess their performance. Table 2 presents the OA, average recognition accuracy, and F1-Score of different methods for each category. It can be observed that machine learning models yield suboptimal recognition results, while deep learning models exhibit superior performance. Notably, large deep models like ResNet-34 and DenseNet achieve OA of 93.19 and 93.07%, respectively, showing the effectiveness of deep learning models on dual-frequency and multi-polarization HRRPs. Our proposed MFFE-Net obtains the highest OA, F1-Score, and AUC, outperforming all other methods with a 5.18% improvement in OA, a 0.0566 improvement in F1-Score, and a 0.066 improvement in AUC over the suboptimal ResNet-34, surpassing the worst-performing SVM by 19.64% in OA, 0.2048 in F1-Score, and 0.2251 in AUC. These demonstrated the effectiveness of our approach in fully learning target features and achieving precise target recognition.

**TABLE 2:** Detailed accuracy results of different types of ESD via several HRRP recognition methods in the first condition.

Method	Car	SUV	Pick-up	Minibus	Bus	Overall accuracy	F1-Score	AUC
Ours	**98.70%**	**99.26%**	**97.59%**	**98.33%**	**98.33%**	**98.37%**	**0.9884**	**0.9905**
SVM	82.59%	97.04%	22.96%	61.30%	95.18%	78.73%	0.7836	0.7654
KNN	92.22%	92.96%	68.70%	79.26%	72.59%	81.15%	0.8132	0.8025
1D-CNN	95.19%	88.15%	76.30%	83.1%	92.96%	87.00%	0.8877	0.8816
LSTMRNN	94.26%	93.52%	81.67%	73.70%	84.81%	85.15%	0.8565	0.8631
SAE	88.70%	87.22%	82.22%	81.30%	98.33%	87.56%	0.8815	0.8785
1D-SCAE	93.52%	87.22%	77.04%	86.30%	93.15%	87.44%	0.8782	0.8780
VGGNet	98.52%	90.00%	85.00%	81.48%	98.89%	90.52%	0.9058	0.9086
ResNet-34	91.85%	95.52%	90.93%	92.22%	97.22%	93.19%	0.9318	0.9245
DenseNet	95.00%	96.11%	88.15%	91.67%	93.70%	93.07%	0.9346	0.9365

The values in bold are the accuracy, F1-Score, and AUC of our method (MFFE-Net).

Furthermore, to verify the robustness of the model, we also compared the pitch angle sensitivity tests of MFFE-NET with other methods. In Table 3, it can be observed that using data with a pitch angle of 28° for training and data with a pitch angle of 30° for testing, all methods show a decrease in recognition accuracy. However, our method still achieves an OA of 90.23%, a F1-Score of 0.9062, and an AUC of 0.9029, outperforming the suboptimal DenseNet with a 3.48% improvement in OA, a 0.0385 improvement in F1-Score, and a 0.0351 improvement in AUC, surpassing the worst-performing SVM by 26.37% in OA, 0.2669 in F1-Score, and 0.2641 in AUC. These demonstrated the robustness of our approach in precise target recognition.

**TABLE 3:** Detailed accuracy results of different types of ESD via several HRRP recognition methods in the second condition.

Method	Car	SUV	Pick-up	Minibus	Bus	Overall Accuracy	F1-Score	AUC
Ours	**89.35%**	**88.72%**	**90.59%**	**89.33%**	**92.71%**	**90.23%**	**0.9062**	**0.9029**
SVM	68.11%	78.25%	25.63%	63.43%	81.92%	63.86%	0.6393	0.6388
KNN	72.18%	70.61%	52.09%	70.54%	62.36%	65.66%	0.6585	0.6549
1D-CNN	84.32%	81.15%	76.62%	78.41%	84.24%	81.08%	0.8126	0.8138
LSTMRNN	80.30%	81.48%	70.77%	73.25%	80.15%	77.22%	0.7713	0.7689
SAE	80.25%	82.26%	75.12%	78.06%	87.34%	81.11%	0.8105	0.8188
1D-SCAE	85.63%	81.12%	78.21%	80.63%	85.03%	82.73%	0.8242	0.8271
VGGNet	90.06%	85.52%	81.10%	76.65%	91.12%	84.86%	0.8525	0.8488
ResNet-34	86.92%	88.60%	81.13%	85.12%	89.42%	86.44%	0.8687	0.8665
DenseNet	86.83%	87.93%	82.86%	86.43%	88.68%	86.75%	0.8677	0.8678

The values in bold are the accuracy, F1-Score, and AUC of our method (MFFE-Net).

#### 3.3.2. Experimental results on RMD

The recognition performance of MFFE-Net with that of traditional target recognition methods were compared. The recognition rate of different methods with each target are shown in Table 4. It can be seen that most deep learning models outperform traditional machine learning methods. Compared with KNN, which has the worst OA, 1D-CNN improves OA by more than 5%, and DenseNet improves OA by 9.46%. This proves the feature extraction and learning capabilities of deep learning-based recognition models. Among the deep models, our proposed MFFE-Net achieved the highest OA, F1-Score, and AUC, surpassing the second-best ResNet34 by 2.01% in OA, 0.0229 in F1-Score, and 0.033 in AUC, outperforming VGGNet and DenseNet more than 3% in OA, and achieving 10% higher than the worst-performing KNN. PA of each target also achieved the ideal recognition performance. Our results show that the proposed method can effectively learn the feature of the target and achieve fine target recognition.

**TABLE 4:** Detailed accuracy results of different types on RMD via several HRRP recognition methods.

Method	Truck	SUV	Van	Overall accuracy	F1-Score	AUC
Ours	**99.03%**	**99.31%**	**98.75%**	**99.03%**	**0.9816**	**0.9873**
SVM	91.20%	95.89%	59.33%	88.60%	0.8625	0.8546
KNN	100%	81.33%	46.94%	87.13%	0.8611	0.8613
1D-CNN	99.41%	98.83%	44.86%	92.24%	0.9021	0.9018
LSTMRNN	98.01%	87.61%	92.92%	93.66%	0.9216	0.9259
SAE	90.95%	96.67%	94.72%	93.27%	0.9252	0.9288
1D-CAE	93.92%	99.94%	84.58%	94.66%	0.9415	0.9411
VGGNet	100%	99.78%	76.53%	96.59%	0.9525	0.9573
ResNet-34	98.89%	94.11%	98.89%	97.02%	0.9587	0.9543
DenseNet	99.93%	98.44%	78.47%	96.59%	0.9439	0.9433

The values in bold are the accuracy, F1-Score, and AUC of our method (MFFE-Net).

Moreover, to better validate the recognition performance of the model on each category, we analyzed the confusion matrix on RMD. In Table 5, we observe that our model achieves a high OA of 99.03% on RMD, among which Truck achieves a PA of 98.75%, SUV achieves a PA of 99.31%, and Van achieves a PA of 99.30%. These demonstrated the effectiveness of our method in achieving accurate recognition. It can be seen that five samples of Truck are misclassified as SUV, which may be because the scattering center characteristics of Truck are close to SUV at some azimuths, increasing the possibility of misjudgment of the model. However, Truck and Van, although similar in shape, can be accurately identified, which further validated the fine recognition capabilities of our model.

**TABLE 5:** Confusion matrix of the FPFR-Net on RMD.

Type	Truck	SUV	Van	PA
Truck	713	5	2	98.75%
SUV	2	715	3	99.31%
Van	7	2	711	99.30%
OA				99.03%

### 3.4. Ablation study and analysis

To further analyze the fusion and recognition effectiveness of MFFE-Net, this paper conducted a series of ablation experiments focusing on two aspects: network modules and loss functions. The first type of ablation experiment examined the effectiveness of submodules (excluding the loss function) within MFFE-Net, including SSE Block, DSE Block, D-AT Block, MFE Block, and REL unit. The second type investigated the validity of loss function, including scattering center loss function and maximum coding rate decline loss function. Except for certain examined components, the rest of the settings remain consistent.

#### 3.4.1. Ablation study of network submodule

The results of the ablation experiments on the MFFE-Net submodule on ESD are presented in Table 6. From lines 1, 2, and 3, it can be observed that using only SSE block achieves an OA of 83.56%, while using only the DSE block achieves an OA of 81.10%. The contribution of the DSE module appears to be relatively small. In the DFIF module, when both SSE and DSE submodules are used simultaneously, an OA of 84.93% is achieved, demonstrating the effectiveness of both SSE and DSE. Furthermore, after adding D-AT and MPF based on line 3, OA improves by 5.38%. Due to D-AT’s attention to information aggregation, the recognition effect has been significantly improved. Finally, with the addition of the REL unit, further improvements in the recognition rates can be observed. This confirms that the REL unit effectively enhances the features. Through comparison, it can be seen that each submodule proposed by us has a positive effect on the recognition task. Our model can fully mine the characteristic information of frequency and polarization dimension, and effectively integrate it to achieve good recognition effect.

**TABLE 6:** Ablation study of submodule.

Version	SSE	DSE	D-AT	MPF	REL	OA
(a)	√	×	×	×	×	83.56%
(b)	×	√	×	×	×	81.10%
(c)	√	√	×	×	×	84.93%
(d)	√	√	√	×	×	88.37%
(e)	√	√	×	√	×	86.89%
(f)	√	√	√	√	×	90.81%
(g)	√	√	√	√	√	93.41%

**FIGURE 7:**
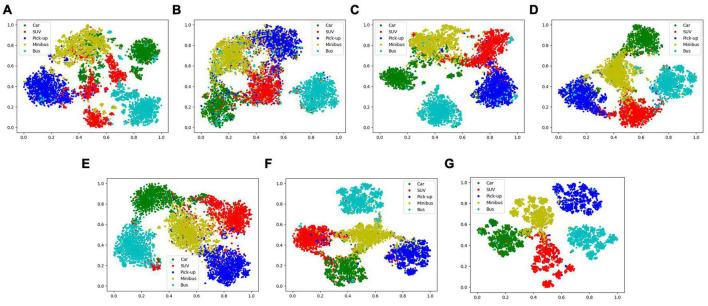
Two-dimensional t-SNE projection of feature vectors extracted from network versions **(A–G)**, corresponds to MFFE-Net version in the ablation study of network submodule.

In order to more intuitively compare the effects of each module, this paper used t-SNE to visualize the feature representation distribution of the test sample, as shown in Figure 7. By comparing (A), (B), and (C), our SSE and DSE both achieve feature separation in the feature space. DSE, in particular, demonstrates a superior effect, proving the significance of dual-frequency HRRP differences in recognition. Moreover, the combined effect of the two blocks surpasses that of a single block, indicating their compatibility when working together. Through a comparison of (C), (D), (E), and (F), the D-AT realized the aggregation of multi-polarization information, and MPF achieved superior fusion results. Furthermore, comparing (G) with other versions confirms the positive impact of all our designed submodules and their ability to achieve collaborative work.

#### 3.4.2. Ablation study of loss function

Based on the submodule ablation experiment, this paper conducted ablation experiments to assess the effectiveness of the loss function, as shown in Table 7. It can be seen from the first and second rows that the network using the scattering loss function increases OA by 2.74%, which proves that the scattering loss function positively contributes to dual-frequency information fusion. Furthermore, it can be seen from the first and third rows that OA increases by 2.37% after adding MCR2 loss function, which proved the separable transformation capability of MCR2 loss. By incorporating both the scattering loss and MCR2 loss, MFFE-Net achieved an OA of 98.37%, thereby validating the effectiveness of the two loss functions proposed in our study.

**TABLE 7:** Ablation study of loss function.

Scatter loss	MCR2 loss	Overall accuracy
×	×	93.41%
√	×	96.15%
×	√	95.78%
√	√	98.37%

**FIGURE 8:**
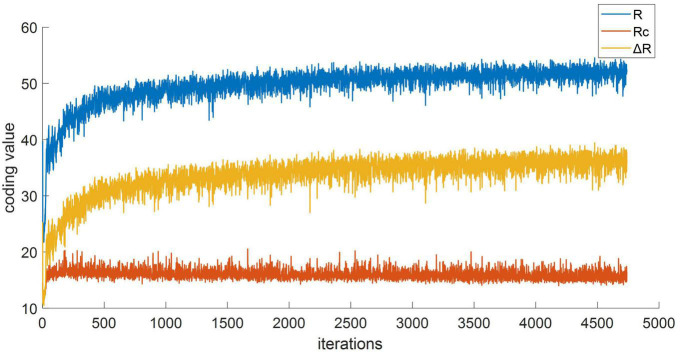
The coding rate curve of feature space.

The effectiveness of MCR2 loss on improving the model’s feature space transformation ability were also explored. The encoding rate serves as a measure of the feature space size: the stronger the feature separability, the higher encoding rate of the whole space *R* and smaller spatial encoding rates within class *Rc*. Figure 8 illustrates the change curve of the value associated with the feature space encoding rate under the aforementioned experimental conditions. It can be seen that the inter-class encoding rate *R* of the feature space gradually increases, while the intra-class spatial encoding rate *Rc* gradually decreases, and the encoding rate difference Δ*R* increases, which indicates explicit expansion of the entire feature space, and each class is being compressed and becoming more compact. Thus, it is easier to achieve accurate target recognition.

## 4. Conclusion

This paper proposes using the brain-inspired neural network (MFFE-Net) to counter the challenging dual-band polarimetric HRRP recognition problem which so far still widely relies on feature extraction within a single dimension and fusion between multidimensional data. Specifically, inspired by the human brain’s multi-dimensional information interaction, selective attention, and hierarchical learning mechanism, the corresponding network modules are designed for multi-frequency scattering information fusion, multi-polarization scattering information fusion, and feature separability enhancement learning, respectively. Experiment results on simulated and measured datasets validate the superiority of the proposed MFFE-Net, which can effectively improve the target recognition accuracy of dual-band polarimetric HRRP. Additionally, ablative studies confirmed the reasonability and effectiveness of submodules and loss functions, which effectively realize the multi-dimensional information fusion and feature separability enhancement.

This work is a preliminary study on the development of dual-frequency and multi polarization fusion recognition. To fully realize their potential, we will further optimize the framework and parameters of the model. Moreover, we consider explicitly embedding the dual-frequency HRRP scattering characteristics into the neural network structure to further improve the interpretability of the model.

## Data availability statement

The datasets presented in this article are not readily available because the dataset is part of ongoing work. All requests to access the datasets should be directed to corresponding author LZ, zhangliang@bit.edu.cn.

## Author contributions

WY and LZ conceptualized the study and proposed the method and analyzed and optimized the proposed network. WY, QZ, and MY conducted the experiments and wrote the original draft of manuscript. LZ, YW, and YL revised the manuscript and provided funding support. All authors have read and agreed to the published version of the manuscript.
